# *In vivo* retinal melanin detection with the calibrated depolarization index in polarization-sensitive optical coherence tomography

**DOI:** 10.1117/1.JBO.29.12.126001

**Published:** 2024-12-19

**Authors:** Mengyuan Ke, Liqin Jiang, Veluchamy A. Barathi, Jocelyn Cheong, Jacqueline Chua, Leopold Schmetterer, Rainer A. Leitgeb, Xinyu Liu

**Affiliations:** a Medical University of Vienna, Center for Medical Physics and Biomedical Engineering, Vienna, Austria; b Singapore Eye Research Institute, Singapore National Eye Centre, Singapore; c Academic Clinical Program, Duke-NUS Medical School, Singapore; d National University of Singapore, Yong Loo Lin School of Medicine, Department of Ophthalmology, Singapore; e Singapore Eye Research Institute, Translational Pre-Clinical Model Platform, Singapore; f SERI-NTU Advanced Ocular Engineering (STANCE) Programme, Singapore; g Nanyang Technological University, School of Chemistry, Chemical Engineering and Biotechnology, Singapore; h Medical University of Vienna, Department of Clinical Pharmacology, Vienna, Austria; i Institute of Experimental and Clinical Ophthalmology, Basel, Switzerland; j Fondation Ophtalmologique Adolphe De Rothschild, Paris, France; k Peking University Health Science Center, Institute of Medical Technology, China

**Keywords:** polarization-sensitive optical coherence tomography, melanin, depolarization

## Abstract

**Significance:**

A data-based calibration method with enhanced depolarization contrast in polarization-sensitive optical coherence tomography (PS-OCT) was developed and demonstrated effective for detecting melanin content in the eye.

**Aim:**

We aim to mitigate the dependence between the measured depolarization metric and the intensity signal-to-noise ratio (SNR) for improved visualization of depolarizing tissues, especially in low SNR regions, and to demonstrate the enhanced depolarization contrast to evaluate melanin presence.

**Approach:**

A function for calibrating the depolarization metric was experimentally derived from the young albino guinea pig, assuming depolarization free in the retina. A longitudinal study of guinea pigs (9 weeks) was conducted to assess the accumulation of melanin during early eye growth. Furthermore, the melanin content of the sub-macular choroid was compared in eyes with light and dark irides involving 14 human subjects in early middle adulthood.

**Results:**

We observed an increase in the improved depolarization contrast, which indicates potential melanin accumulation in the early eye development with age in the pigmented guinea pig eyes. We found a significant difference in melanin content between human eyes with light and dark colors.

**Conclusions:**

Our proposed calibration method enhanced the visualization of depolarizing structures in PS-OCT, which can be generalized to all kinds of polarization-sensitive imaging and can potentially monitor melanin in healthy and pathological eyes.

## 1 Introduction

Melanin granules are pigmented structures of non-spherical particles with a radius of ∼1  μm of different shapes, richly distributed in the retinal pigmented epithelium (RPE) of human eyes. Changes in the concentration and distribution of melanin have been observed during normal aging^1^ or under disease conditions such as albinism^2^ and age-related macular degeneration.^3^^–^^5^ In addition, melanin-rich contented tissues may reduce the transscleral retinal and vitreal drug delivery efficiency via binding drugs such as celecoxib.^6^ Hence, developing non-invasive methods to evaluate the melanin content and distribution in the eye may be important for diagnosing and managing various ocular diseases.^7^^,^^8^ Polarization-sensitive optical coherence tomography (PS-OCT) is the functional extension of OCT and can measure both depth-resolved reflectivity and the polarization states of the light backscattered from tissues.^9^^,^^10^ PS-OCT was first used to observe the scrambling of polarization in the highly pigmented RPE layer,^11^^,^^12^ and later was proved to be useful in evaluating the intactness of RPE *in vivo*.^4^^,^^13^^–^^16^ The RPE layer can be distinguished from other retinal layers under the depolarization contrast of PS-OCT, which is likely attributed to the anisotropic scattering from the dense and non-spherical particles of different sizes of the melanin granules that are richly distributed in the RPE. Depolarization-related metrics have been shown to monotonically increase in response to concentration and sizes in melanin suspension.^10^^,^^13^^,^^17^ Depolarization contrast can be calculated from the PS-OCT measurements based on various metrics, including the degree of polarization uniformity (DOPU), the degree of polarization, and the depolarization index.^15^ A recent comprehensive comparison of entropy, depolarization index, and DOPU was reported based on a melanin suspension study and further demonstrated in the human RPE.^16^ However, further investigation into the depolarization measurement of animal and human eyes across different ages and races is needed to interpret the PS-OCT depolarization as a clinically endogenous contrast.

In this paper, we experimentally measured the empirical relation function between the depolarization metric and the intensity signal-to-noise ratio (SNR) and developed a method to calculate the introduced calibrated depolarization index (CDI) to mitigate the correlation between the measured depolarization metric and the intensity SNR. We hypothesize that a high concentration of melanin in the choroid will exhibit a stronger depolarizing contrast revealed by the high CDI. Using the proposed CDI, we longitudinally investigated the melanin accumulation in young guinea pig eyes up to 9 weeks old. In addition, we compared melanin content in Asian and Caucasian human eyes with light and dark iris colors. The study has two novelties. First, we observed the accumulation of melanin in the choroid and profiled the CDI change associated with the growth of the eye in pigmented guinea pigs from birth to 9 weeks old. Second, we found that the depolarization signal was significantly different in Asian and Caucasian eyes in the submacular choroid region, which suggests additional cautions should be made when using the depolarization metrics to evaluate the retinal pigmented structures.

## 2 Materials and Methods

### 2.1 PS-OCT System

The PS-OCT system used in this study was previously developed in our lab, named triple-input PS-OCT (TRIPS-OCT).^18^^,^^19^ In brief, it used a polarization modulator to produce three Poincaré-space-orthogonal states to illuminate the sample. Each B-scan location was repetitively scanned three times under the modulation of a particular polarization state. A swept source was employed with a center wavelength of 1060 nm, a bandwidth of 100 nm, and a sweep rate of 200 kHz. A conventional balanced polarization diversity detection unit was used to detect the fringes in the two polarization channels after the interference occurred between the backscattered and reflected light from the sample and the reference arms. For human eye imaging, the beam size at the pupil was 0.67 mm, corresponding to an optical lateral resolution of 44  μm in a normal human eye with an axial length of 23 mm. For guinea pig eye imaging, lateral optical resolution is 15.3  μm in a normal guinea pig eye with an axial length of 8 mm.

### 2.2 Guinea Pigs

Five guinea pigs (Elm Hill Labs, Chelmsford, Chelmsford, Massachusetts, United States), four pigmented and one albino, were bred on-site and imaged weekly from October 20, 2020, to December 26, 2020. The pigmented guinea pigs were imaged weekly until they were 9 weeks old. The albino guinea pig was imaged weekly until 7 weeks old. The animals were reared under a 12-h light/12-h dark cycle with lights on at 08:00 in the animal-center facilities. The animals had free access to standard food and water. The use of animals for these studies was approved by the Institutional Animal Care and Use Committee of SingHealth (AAALAC Accredited; 2018/SHS/1441, IACUC 1290). All procedures adhered to the ARVO Statement for the Use of Animals in Ophthalmic and Vision Research.

### 2.3 Guinea Pig Eye Imaging

Prior to imaging, guinea pigs were injected with 27-mg/kg ketamine and 0.6-mg/kg xylazine based on their body weight for anesthetic, and the eyes were dilated with 1% tropicamide (Alcon Laboratories, Inc., Fort Worth, Texas, United States) and 2.5% phenylephrine (Bausch and Lomb Pharmaceuticals, Inc., Tampa, Florida, United States) ophthalmic solutions. A volumetric scan of a 22-deg field of view was performed on each eye centering at the optic nerve head (ONH). The volume comprised 1000 (fast scanning axis) by 3000 (slow scanning axis) A-lines. Note that due to the polarization modulation, the sampling number was factored by three along the slow scanning direction.

### 2.4 Scanning Electron Microscopy Analysis

One pigmented and one albino guinea pig at 15 weeks old were euthanized, and their eyes were enucleated and immersed in a 2% glutaraldehyde and paraformaldehyde cocktail overnight at 4°C. Tissue patches located 2 mm from the optic nerve were dissected and treated with tannic acid. To compare the structures of the choroid and sclera between albino and pigmented guinea pigs, stitched images were obtained from 500-nm sections using an SEM system (FEI QUANTA 650 FEG).

### 2.5 Depolarizing Metric Index Calculation

To account for the variations in DOPU that are due to input polarization states,^17^ the samples are illuminated with three controllable orthogonal polarization states in TRIPSOCT.^18^ Furthermore, spectral binning was employed when the DOPU was initially calculated to eliminate the randomness caused by the polarization dispersion mode.^20^ Spectral binning^20^ was performed by evenly dividing the fringes into nine partially overlapping sub-spectral bins. Stokes vectors were reconstructed from the fringes in each bin. Spatial averaging was performed on both the Stokes vectors and the intensity maps. The averaging kernel window size was set as 9×9×9  voxels. DOPU maps of sub-spectral bins were calculated as the quotient between the second-order norm of the spatially averaged Stokes vectors and the averaged intensity. The overall DOPU map was obtained by averaging 27 sub-spectral bin DOPU maps, including nine maps from three modulated polarization states. The depolarizing metric index (DI) was calculated by DI=1−DOPU.

### 2.6 Calibrated Depolarization Index

Following the numerical analysis in PS-OCT^21^ showing the dependency correlation of DOPU with SNR in the presence of increasing noise, we experimentally determined the relation function in the absence of polarization scrambling predominately caused by melanin in albino guinea pig *in vivo*. We assume that this determined relation function can mitigate the dependence to achieve optimal performance similar to the noise-corrected DOPU metric so that the proposed depolarization metric is a useful correlation indicator to assess the melanin contents. The mixing polarization states caused by unknown scattering before the choroid would make the empirical relation function broader.^22^ In the animal model, we can clearly define an empirical function derived from the segmented tissue above the choroid. We observed a strong correlation between the DI and the intensity SNR. This correlation may be caused by intensity noise and speckle, which was coupled into the measured Stokes vectors and, therefore, contributed additional noise to the DI calculation. To mitigate the correlation between DI and the intensity SNR so that the DOP can be reliably used to investigate the depolarization property of the underlying tissues, we propose the CDI, which was calculated using an empirical calibration method to decouple the intensity SNR and the DI as follows. First, we segmented the retina, defined as the retina tissue above RPE in the posterior eye, of the B-scan in a typical albino guinea pig eye, and subsequently fitted a relation function between the SNR and DI, noted as f(SNR). Given the absence of melanin granules in the retina, we assumed that this relation function DI=f(SNR) always held under the condition of no physically depolarizing structures present in the tissue. If physical depolarization existed in the tissue, the DI values should be higher than predicted by the function f(SNR). Second, we define CDI=DI−f(SNR) and further rectify the CDI value to the range of [0, 1] by

$$\mathrm{CDI}=\{\begin{array}{ll} 0, & \mathrm{DI}<0\\ \mathrm{DI}-f(\mathrm{SNR}), & 0\leq \mathrm{DI}\leq 1\\ 1, & \mathrm{DI}>0. \end{array}$$ (1)

Assuming CDI at a low SNR region (SNR<5  dB) does not characterize tissue-induced depolarization effect, due to the excessive noise. CDI is manually set to 0, when they exceed the boundary value. Hence, the proposed CDI is consistently rectified within [0, 1] in the region where SNR≥5  dB.

### 2.7 Human Imaging

Fourteen healthy volunteers (age 35±8 years; 4 males and 10 females; 5 Caucasian and 9 Asian) with no history of systemic or ocular diseases participated in the study at the research clinic of the Singapore Eye Research Institute from February 18, 2021 to May 14, 2021. Triple input PS-OCT scans were performed on both eyes with a scan of 1000×700×3 A-lines in a region corresponding to a 12 mm by 9 mm rectangular centered at the fovea. The pupil was dark-adapted before imaging, and no dilation was performed. The study was conducted in accordance with the Declaration of Helsinki and approved by the Institutional Review Board of Singapore Health Services (protocol no R1617/14/2019, approved on April 13, 2019).

## 3 Results

### 3.1 Correlation between DI and SNR

**Fig. 1:**
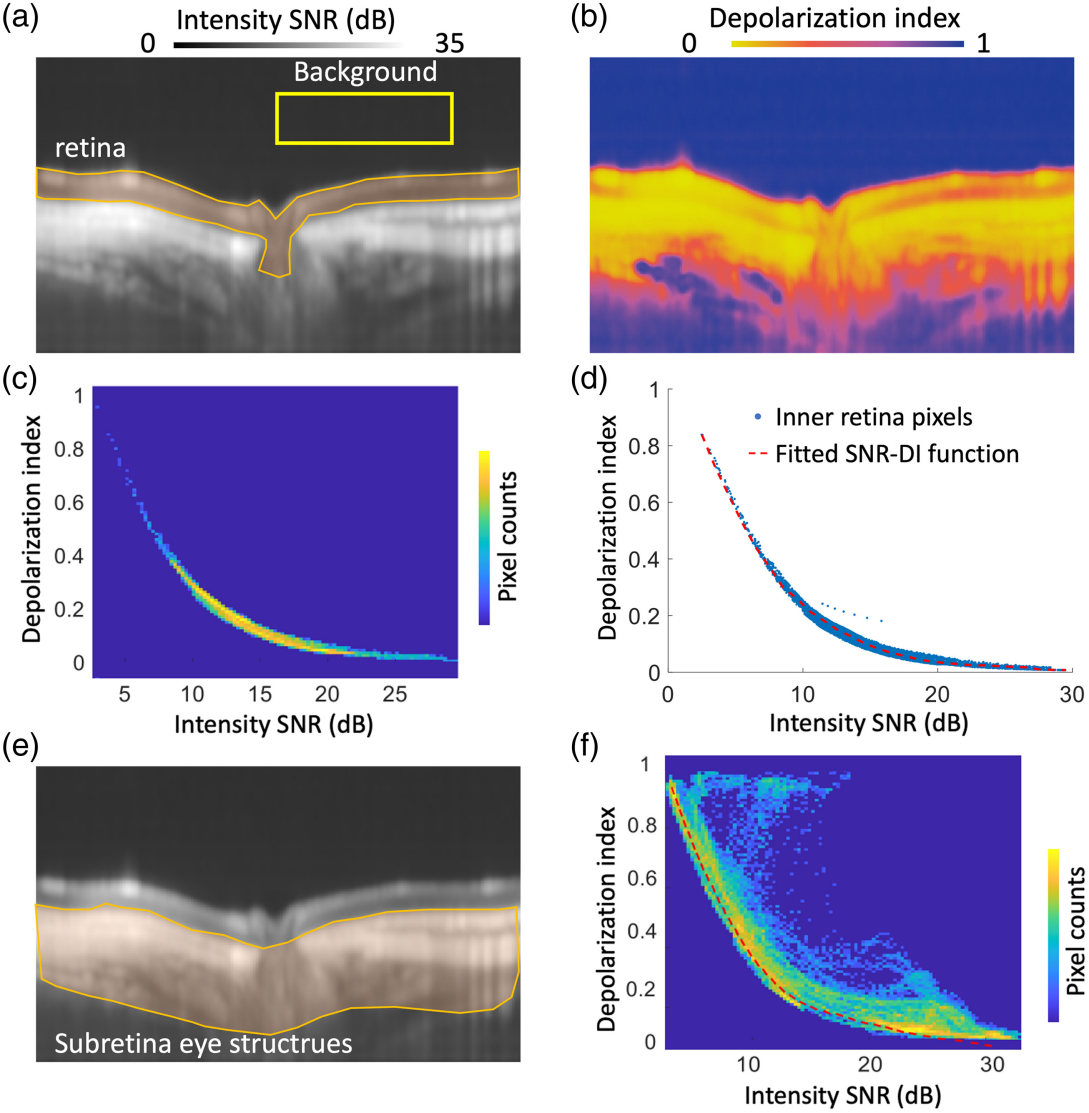
(a) Representative image of intensity SNR calculated with a selected background (yellow box) as noise floor; (b) representative image of reconstructed DI; (c) segmented retina DI versus the 2D histogram of intensity SNR; (d) fitted SNR-DI function in the retina; (e) segmented sub-retina segmented region (orange region) overlapped in the intensity SNR; (f) segmented sub-retina DI versus the 2D histogram of intensity SNR.

To obtain the correlation between DI and SNR, a representative albino guinea retina was imaged centering at ONH by TRIPS-OCT *in vivo*. We used the mean intensity value of an area above the retina [yellow box in Fig. 1(a)] as the noise floor and computed the intensity SNR [Fig. 1(a)]. All image data were acquired close to zero delay to minimize the variance in the noise power. The noise level, chosen as the mean noise of the selected window region, is calculated for each acquired volume. We assume that the noise level is similar across depth after proper k-sampling; however, in the future, the depth-dependent noise level should be considered when data acquired are away from zero delay in the presence of increasing noise power.^23^ We then reconstructed the depolarizing metric index (DI) map [Fig. 1(b)]. As the retina of the albino guinea pig contains no melanin granules, the relation of DI and SNR can approximate that of a depolarization-free medium. From the manually segmented retina volume [the corresponding region of interest is indicated in the B-scan: orange area in Fig. 1(a)], we plotted the distribution of SNR-DI scatters in a 2D histogram [Fig. 1(c)]. We observed that the DI-SNR scatters were nicely distributed on a monotonic decreasing trace. We then fitted an empirical spline function (piecewise polynomial was fit with 34 pieces and order of 4) from SNR to DI [Fig. 1(d)]. To evaluate the SNR-DI correlation in depolarizing tissues, we further segmented the sub-retina structures [Fig. 1(e)], including the choroid, sclera, and posterior eye tissues. We plotted the SNR-DI scatter histogram [Fig. 1(f)] and observed that SNR-DI scatters appeared above the fitted SNR-DI function, suggesting that depolarizing effects exist in the underlying tissues.

### 3.2 CDI Evaluation of Melanin in Guinea Pigs

**Fig. 2:**
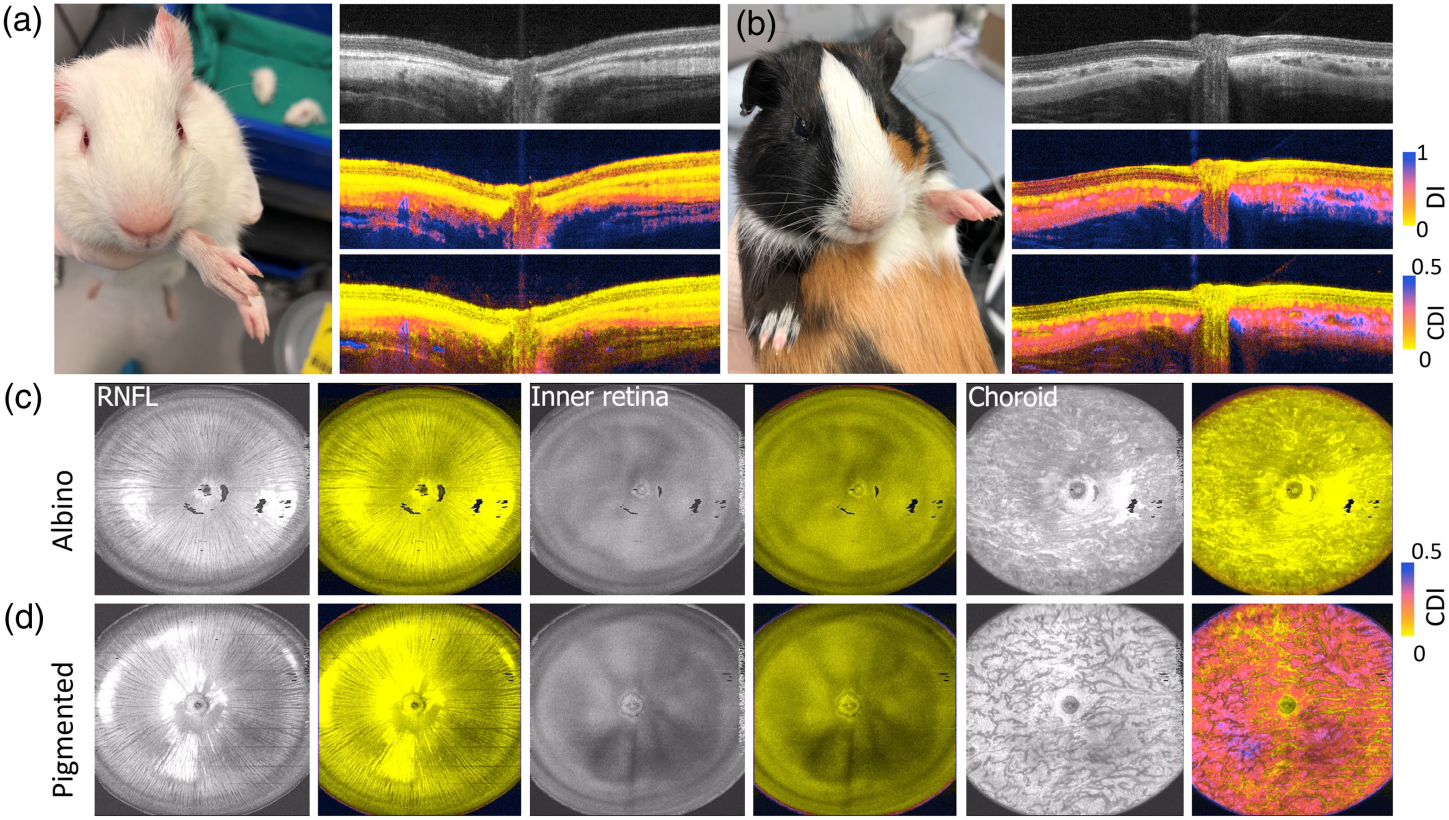
(a) Representative B-scan of intensity, DI, and CDI for albino guinea pig retina; (b) representative B-scan of intensity, DI, and CDI for pigmented guinea pig retina; (c) reconstructed en face maps of CDI corresponds to the segmented volumes of RNFL, retina, and choroid for albino guinea pig; (d) reconstructed en face maps of CDI corresponds to the segmented volumes of RNFL, retina, and choroid for pigmented guinea pig.

To test whether our proposed CDI can improve the contrast of depolarization and represent the melanin distribution in the eye, we compared the eyes of an albino [Fig. 2(a)] and a pigmented [Fig. 2(b)] guinea pig (8 weeks, female). In the comparison, we observed that the CDI better represented the depolarized regions in the retina than the DI. In the albino eye, the CDI was low in the choroid and sclera. By contrast, in the pigmented eye, the CDI was high in the choroid and choroid-sclera transition areas. We further rendered the en face maps from 3D scans of the albino and the pigmented eyes. In the en face planes, we observed that the retinal nerve fiber layer (RNFL) and retina were similarly low in CDI between the albino [Fig. 2(c)] and the pigmented [Fig. 2(d)] eyes. On the contrary, in the choroid layer, the CDI map of the pigmented eye was obviously higher than that of the albino eye.

### 3.3 SEM Analysis of Melanin

**Fig. 3:**
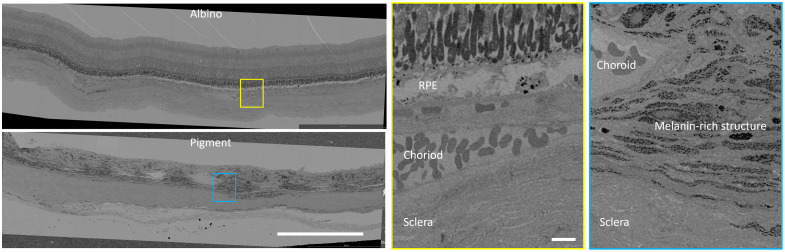
Scanning electron microscope images for the albino and the pigmented guinea pig retina fundus; scale bar: 300  μm. Zoom-in views for albino guinea pig interface between the retina and choroid (selected yellow box) and pigmented guinea, the interface between the choroid and sclera (selected blue box); scale bar: 10  μm.

To further understand the melanin distribution in the albino and pigmented guinea pig eyes, we used SEM to analyze the sliced tissues from one albino and one guinea pig (15-week female) eye. In the SEM scans, we clearly observed the individual melanin granules in the tissue. In the albino eyes, we observed that there were no melanin granules in the choroid and upper sclera. However, in the pigmented eye, melanin granules were richly distributed in the choroid and even more concentrated in the transition area between the choroid and sclera (zoom-in view of the blue box in Fig. 3). These SEM analyses were qualitatively consistent with CDI maps measured by the TRIPS-OCT.

### 3.4 Longitudinal Melanin Observation with CDI during Eye Growth in Guinea Pigs

**Fig. 4:**
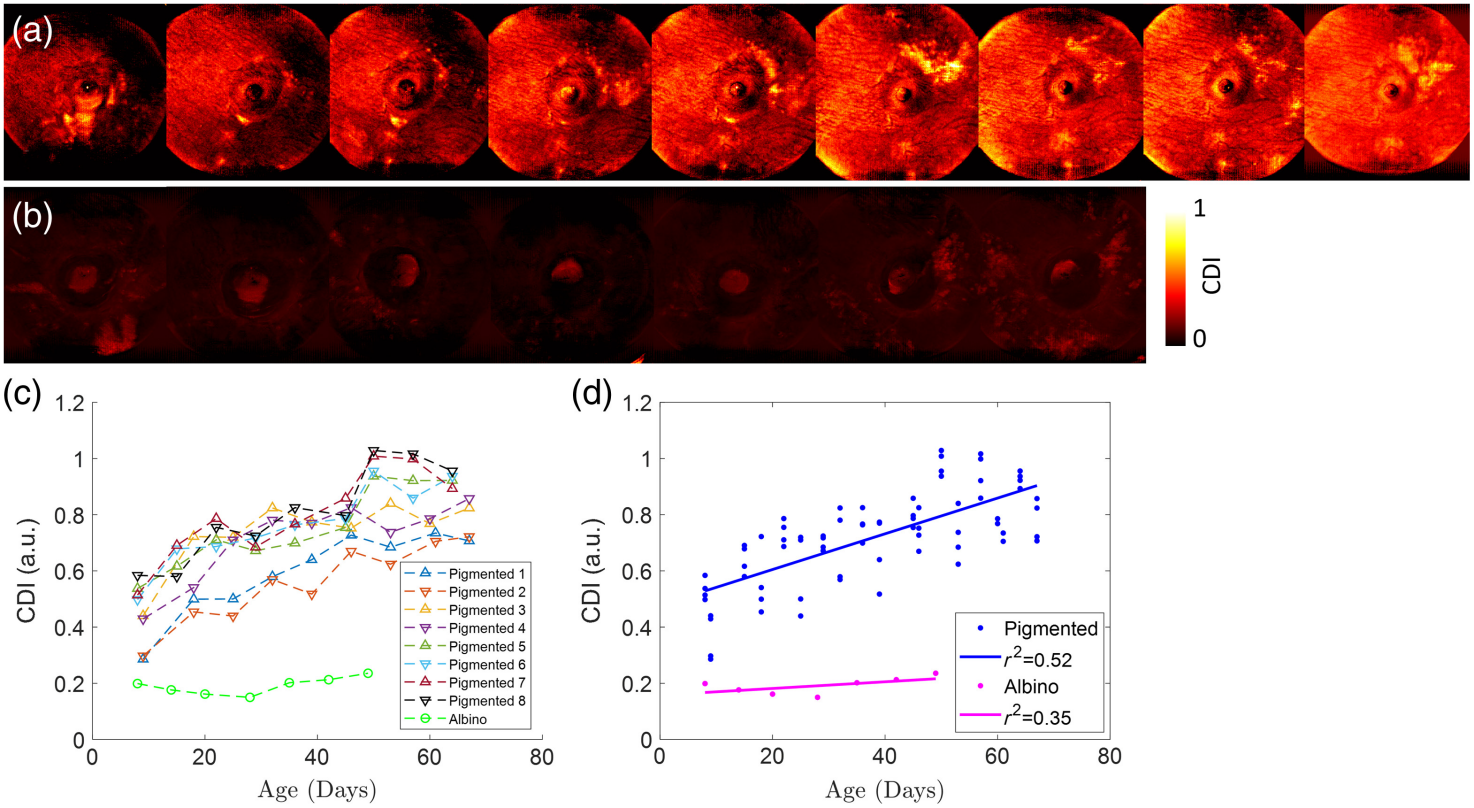
(a) Representative en face maps of CDI with age for a pigmented guinea pig from week 1 to 9; (b) representative en face maps of CDI with age for an albino guinea pig from week 1 to 7; (c) individual eyes’ mean CDI with age; (d) linear regression analysis of pooled data for albino and pigmented guinea pig eyes.

To investigate the melanin variation during early eye growth in guinea pigs, we longitudinally imaged four pigmented guinea pigs weekly aged 1 to 9 weeks. To compare the pigmented and albino eyes, we also imaged 1 albino guinea pig weekly for 7 weeks. En face maps of CDI, rendered by an average of CDI with intensity SNR > 5 dB along the depth direction, showed the increase with age in the pigmented eye [Fig. 4(a), one pigmented eye age from week 1 to 9], and small variation in the albino eye [Fig. 4(b), one albino eye age from week 1 to 7]. We then averaged the en face CDI maps to obtain the mean CDI values. We observed that the mean CDI in the pigmented eyes [Fig. 4(c)] increased, and the mean CDI values were significantly lower in the albino eye than in the pigmented eyes. Using linear regression, we found that the mean CDI increased by 0.0469 per week from the age of week 1 to 9 in the pigmented eyes [Fig. 4(d)], indicating possible accumulation of the pigmented structures during the early development of the guinea pig eyes.

### 3.5 Dark Versus Light-Pigmented Eyes in Humans

**Fig. 5:**
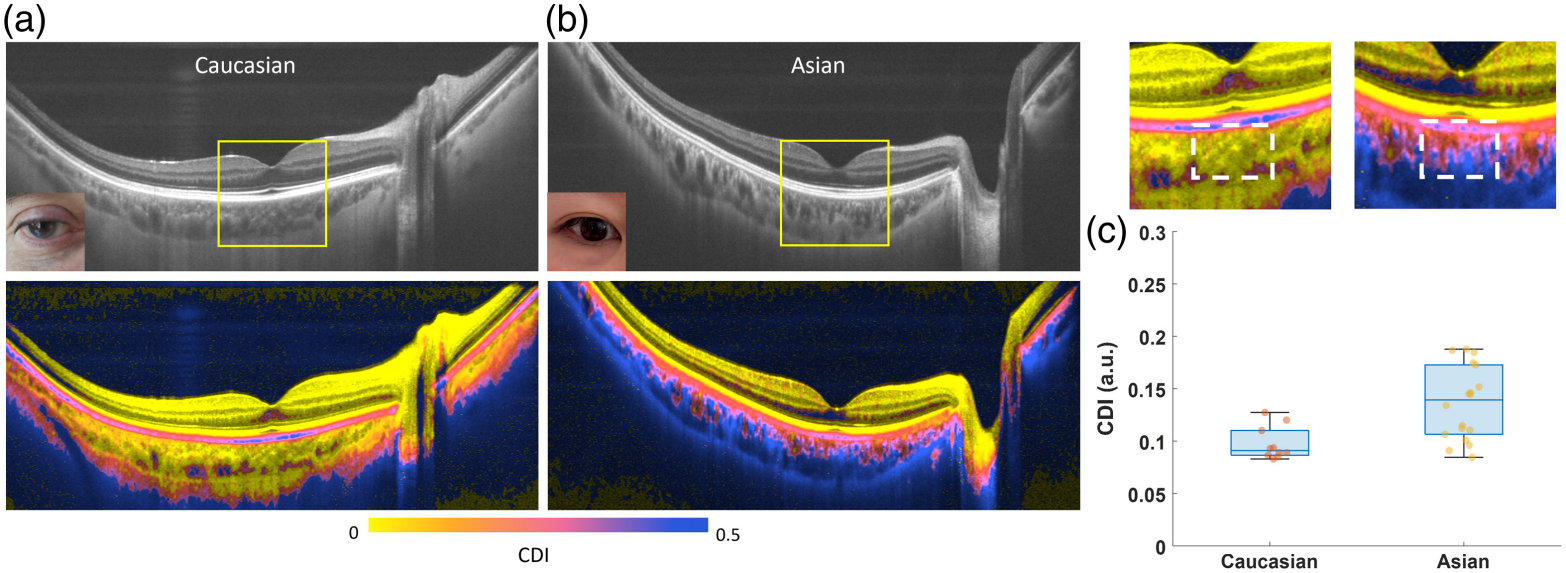
(a) Representative cross-sectional B-scan intensity and CDI images of a Caucasian eye; (b) representative cross-sectional B-scan intensity and CDI images of an Asian eye; (c) zoom-in views of selected region [yellow box in panels (a) and (b)] and bar plots of mean CDI computed from selected submacular choroid region (white dash boxes), comparing 10 Caucasian with 18 Asian eyes.

To validate if CDI is a useful biomarker to evaluate the pigmentation differences in the posterior of human eyes, we imaged 14 healthy participants with light and dark iris colors, including 10 Caucasian (mostly light brown or blue iris) and 18 Asian (mostly black or dark brown iris) eyes. We found that in both the Caucasian and Asian eyes, the RPE layer was highly depolarizing, exhibiting a clear separation from the upper retinal structures in CDI maps [Figs. 5(a) and 5(b)]. However, below the RPE, the choroid in Asian eyes showed higher CDI than that in Caucasian eyes. To further compare the difference, we calculated the averaged CDI value in a submacular choroid region, made of 200×200  pixels below the fovea pit [white dash boxes in the zoom-in views of the selected yellow box region in Figs. 5(a) and 5(b)]. We found that the submacular choroidal CDI was significantly lower in the Caucasian eyes with mostly light brown or blue iris (0.097±0.015) than in the Asian eyes with mostly black or dark brown iris (0.14±0.04, p<0.001) [Fig. 5(c)].

## 4 Discussion and Conclusion

Following the observation that the PS-OCT-measured depolarization metrics, including DOP-uniformity,^12^ depolarization index,^17^ and entropy,^16^ are strongly affected by SNR,^21^^,^^24^ we proposed an empirical method to mitigate the dependence between the measured depolarization metrics and the intensity SNR to improve the characterization of the depolarizing events caused by the underlying tissues. Our assumption is that this empirical method can mitigate the dependency of depolarization metrics on SNR to achieve similar performance as the noise-model-based method previously proposed.^21^ We anticipate that the calibrated metrics profile can then reflect the influence of scattering on the measured depolarization, and we expect the metric to exhibit a trend that closely follows changes in scattering properties, as shown in Ref. 24. Our experimental results on our various retinal samples show that this is a promising method to detect choroidal pigmentation using PS-OCT.

In guinea pig results, CDI maps increased with age in the young pigmented guinea pigs’ eyes, indicating that the melanin contents change during early eye growth, which may be related to the development of the vision system. The observed CDI change with melanin contents *in vivo* agrees with other studies,^13^^,^^16^^,^^21^ reporting a monotonic increase of DOPU or entropy with increasing melanin concentration in phantom, implying an increase of melanin granules in pigmented eyes, and might suggest a possible active melanin synthesis in the early eye development, which was observed in mouse iris,^25^ and in human.^26^ As a preliminary investigation, we selected albino and pigmented guinea pig retinas to examine the pigmentation difference presented by CDI as two distinct cases. In future studies, we intend to expand the animal sample size and incorporate pigmentation concentration groups to ascertain the efficacy of the proposed method in demonstrating significant differences. In our human study, we used the CDI contrast to evaluate the melanin distribution in the posterior eye. Evaluation of the pigmented structures in human eyes is important in diagnosing several eye diseases.^7^^,^^8^ Various imaging technologies, including OCT,^12^^,^^27^^,^^28^ auto-fluorescence,^29^ photoacoustic,^30^^,^^31^ fundus photography,^32^ and reflectometry,^33^ have been proposed^34^^,^^35^ to assess melanin. PS-OCT has the advantage of depth-resolved imaging and can characterize melanin non-invasively using light depolarization as a specific contrast and has been reported to interrogate RPE mostly.^14^^–^^16^ With multiple modulated polarization states of input light centered at 1060 nm, our improved depth imaging revealed the melanin contents in both RPE and choroid. In the comparison study, we found that CDI was significantly higher in dark-color eyes (most Asian) than in light-color eyes (most Caucasian) in the submacular region. We hypothesized that this may correlate with the presence of higher pigmentation structures and predominantly melanin content in early middle-aged Asian human subjects than Caucasians. However, it is important to exercise caution when distinguishing between melanin and other retinal-pigmented structures, such as lipofuscin, using depolarization metrics. As this is a pilot study designed to assess the feasibility and efficacy of the proposed method, a future study involving a different age group may further validate this point. A few studies have proposed using depolarization contrast to evaluate melanin content in the choroid.^36^ Though choroidal melanin concentrations of different iris colors have been evaluated *ex vivo*,^37^^,^^38^ to the best of our knowledge, it has not been investigated whether the melanin content is significantly different in eyes with different iris colors using PS-OCT *in vivo*. The observed differences in melanin content may provide critical insights for clinical treatments of patients with different eye colors, for instance, concerning laser therapy dosimetry for individuals.^39^

One limitation of the study is that the CDI method is not calibrated against a gold standard, so it may not be quantitatively or linearly related to the concentration of melanin. Further study is required to include controllable melanin phantom solutions to evaluate the performance of the correlation between calibrated depolarization metrics and melanin concentration, to investigate whether a linear dependence can be found, as has been reported in other studies.^13^^,^^16^ Moreover, future experiments could be designed to incorporate induced additive noise into the sample to ascertain whether the proposed method can attain comparable performance to that of the numerical method.^21^

**Fig. 6:**
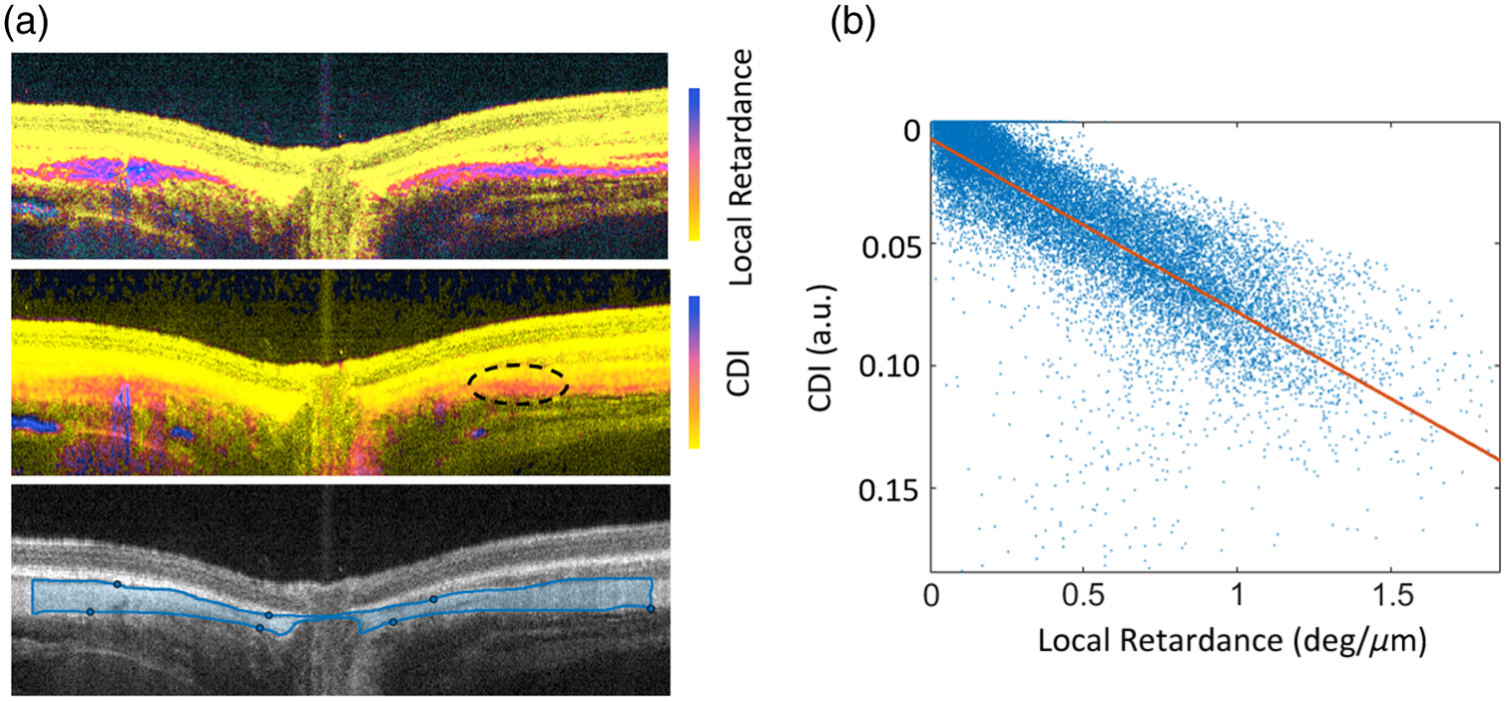
(a) Birefringence, CDI, intensity B-scan of an albino guinea pig retina; (b) correlation between retardance and CDI in the segmented region (blue box).

Another limitation is that, in the albino guinea pig’s eyes, we observed weak depolarization signals in the sclera, where there is no melanin as conformed in the SEM analysis. We believe that the weak depolarization signal may come from the strong birefringence of the tissues because the birefringence can randomize the polarization locally within the tissue. To further analyze the relationship of birefringence with the CDI, we reconstructed the birefringence map of a typical scan in the eye of an albino guinea pig (Fig. 6). We manually segmented the sclera [blue region in Fig. 6(a)] and plotted the depolarization and birefringence values at each pixel location in the sclera. As expected, the birefringence and CDI showed a high correlation [Fig. 6(b)], confirming that tissue birefringence can induce weak depolarization signals. This has to be taken into consideration when interpreting the CDI images.

Our study presented the use of experimentally measured data to approximate the correlation between the depolarization property and the intensity SNR in a nearly depolarization-free medium, thus revealing the depolarization of the tissue via calibrating the DI measurement in PS-OCT with the measured SNR-DI function. We have shown that CDI can assess depolarized structures in both guinea pigs and humans, especially at lower SNR. The preliminary study shows that the depolarization metric derived from the Müller matrix has the potential to be used as a meaningful measure for evaluating depolarizing tissues in a clinical context. Although we acknowledge that the complex underlying physical mechanisms of depolarization is not fully elucidated, we are concentrating on the potential of this metric as a biomarker. We have established a connection between this metric and melanin content in animal models and clinical environments, indicating its potential clinical importance. The calibration method is simple to establish and can be easily applied to all PS-OCT modalities, potentially improving depolarization tissue-specific imaging for enhanced clinical analysis.

## Data Availability

The authors confirm that the data supporting the findings of this study are available within the article upon reasonable request.
